# Survivin-Sodium Iodide Symporter Reporter as a Non-Invasive Diagnostic Marker to Differentiate Uterine Leiomyosarcoma from Leiomyoma

**DOI:** 10.3390/cells12242830

**Published:** 2023-12-13

**Authors:** Natalia Garcia, Mara Ulin, Qiwei Yang, Mohamed Ali, Maarten C. Bosland, Weiqiao Zeng, Liaohai Chen, Ayman Al-Hendy

**Affiliations:** 1 Department of Surgery, University of Illinois at Chicago, Chicago, IL 60607, USA; natalia.garciaft@gmail.com (N.G.); maraulin1989@hotmail.com (M.U.); yangq@bsd.uchicago.edu (Q.Y.); mohamed.ali@bsd.uchicago.edu (M.A.); wzeng9@uic.edu (W.Z.); lhchen@uic.edu (L.C.); 2 Greehey Children’s Cancer Research Institute, The University of Texas Health Science Center, San Antonio, TX 77030, USA; 3 Department of Obstetrics and Gynecology, Mount Sinai Hospital, Chicago, IL 11537, USA; 4 Department of Obstetrics and Gynecology, University of Chicago, Chicago, IL 60637, USA; 5 Clinical Pharmacy Department, Faculty of Pharmacy, Ain Shams University, Cairo 11566, Egypt; 6 Department of Pathology, University of Illinois at Chicago, Chicago, IL 60607, USA; boslandm@uic.edu

**Keywords:** uterine mass, uterine leiomyosarcoma, uterine leiomyoma, PET/CT scan

## Abstract

Leiomyosarcoma (LMS) has been challenging to diagnose because of limitations in clinical and radiographic predictors, as well as the lack of reliable serum or urinary biomarkers. Most uterine masses consist of benign leiomyoma (LM). However, it is currently a significant challenge in gynecology practice to differentiate LMS from LM. This inability poses grave consequences for patients, leading to a high number of unnecessary hysterectomies, infertility, and other major morbidities and possible mortalities. This study aimed to evaluate the use of Survivin-Sodium iodide symporter (Ad-Sur-NIS) as a reporter gene biomarker to differentiate malignant LMS from benign LM by using an F18-NaBF_4_ PET/CT scan. The PET/CT scan images showed a significantly increased radiotracer uptake and a decreased radiotracer decay attributable to the higher abundance of Ad-Sur-NIS in the LMS tumors compared to LM (*p* < 0.05). An excellent safety profile was observed, with no pathological or metabolic differences detected in Ad-Sur-NIS-treated animal versus the vehicle control. Ad-Sur-NIS as a PET scan reporter is a promising imaging biomarker that can differentiate uterine LMS from LM using F18-NaBF_4_ as a radiotracer. As a new diagnostic method, the F18 NaBF_4_ PET/CT scan can provide a much-needed tool in clinical practices to effectively triage women with suspicious uterine masses and avoid unnecessary invasive interventions.

## 1. Introduction

Annually, around twenty-six million patients present with uterine masses [1,2,3]. Uterine leiomyoma (LM) is the most common benign female pelvic neoplasm [4,5]. By age 50, around 80% of African Americans and 70% of Caucasian women will have LM [6]. Uterine leiomyosarcoma (uLMS) is a highly aggressive gynecology malignant mesenchymal tumor of myometrial smooth-muscle derivation. It is the most common type of uterine sarcoma, with low survival rates, representing 60% and accounting for approximately 1–2% of uterine malignancies [7,8,9,10]. uLMS is less common than LMs and has a worse prognosis [11].

Both LM and LMS manifest as focal masses in the uterus that can be accompanied by abnormal uterine bleeding, pelvic pressure, and/or pain. The clinical evaluation of a pelvic mass includes a complete medical history and an abdominal and pelvic examination. Sadly, there is frequently no discernible difference between the clinical characteristics of uterine sarcomas and benign LM. A pelvic ultrasound is typically ordered as the first imaging test for patients with a pelvic tumor. Imaging studies frequently lack the ability to demonstrate any difference between LMs and uterine sarcomas, which are both localized tumors inside the uterus that may have central necrosis. Despite these drawbacks, this is part of the first-line workup. Endometrial sampling is frequently performed in patients with abnormal uterine bleeding and pelvic mass to identify neoplasia. Still, only 33 to 68 percent of individuals with uterine sarcomas receive a diagnosis of sarcoma from an endometrial biopsy. 

Treatment options for LM include expectant, medical, interventional, and surgical therapies. Treatment objectives should be specified for each patient, taking into account the primary symptomatology, such as bleeding and bulk symptoms, as well as the desire to preserve the uterus and future fertility [12]. Evaluation includes risk stratification and the use of imaging, cervical cancer screening and endometrial tissue sampling to identify malignancy [13]. 

When surgical treatment is desired, treatment options include myomectomy and hysterectomy. Whenever possible, the least invasive route is advised. Minimal invasive surgery, either laparoscopic or robotic surgery, compared to laparotomy, is associated with less postoperative pain, shorter hospitalization, a lower risk of postoperative fever, and a faster return to work [13]. When utilizing the least invasive procedure, uterine morcellation is occasionally used to retrieve the uterus or LM. Nevertheless, it is important to be aware of the cancer risk because women who undergo minimally invasive surgery and have undiagnosed uLMS may be more likely to experience an increase in morbidity due to the spread of cancer cells during the procedure [14,15].

The true prevalence of uLMS is unknown [16,17]. Based on the 2017 agency for healthcare research and quality report, which used the largest dataset to determine the estimate of the prevalence of uLMS, the risk of unexpected uLMS in surgeries performed for symptomatic LM can range from 1 in 770 surgeries to less than 1 in 10,000 surgeries [18]. In addition, diagnostic biomarkers to distinguish benign LM versus malignant LMS have not been established yet. Although the levels of serum CA125 and LDH were elevated in uLMS, the sensitivity and specificity were low [19]. Currently, no diagnostic tool (laboratory test or imaging study) can provide a differential diagnosis between LM and uLMS before surgical intervention [7,20,21]. LM and LMS tumors present with similar symptoms (abnormal uterine bleeding, pelvic mass, and pelvic pain). By contrast, the clinical outcome for these tumors is entirely different [22]. 

A major challenge for gynecologists is to differentiate LM from LMS before surgery. At present, there are no pathognomonic signs, symptoms, reliable radiographic predictors, or biomarkers (serum or urinary markers) to differentiate between these two conditions [7,20,21,23,24,25]. 

The FDA has recommended limiting the use of laparoscopic power morcellation in women with suspected or confirmed cancer undergoing gynecological surgeries. Diagnosing uLMS before surgery can be crucial for an improved patient outcome, by providing a better treatment approach. The LM patient can benefit from an early differential diagnosis to avoid unnecessary surgeries, or can choose non-invasive surgery. In contrast, the uLMS patient can have a timely intervention for this aggressive disease.

**Figure 1:**
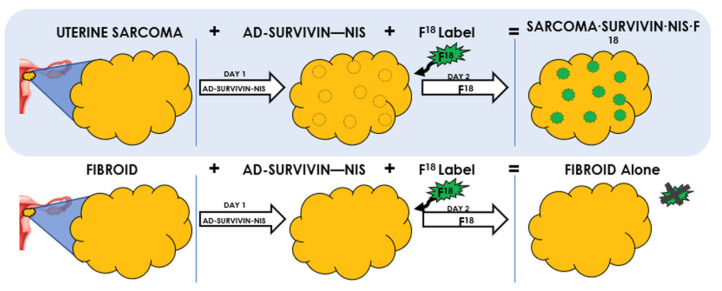
Adenovirus Survivin-Sodium Iodide Symporter (Ad-Sur-NIS) System. It is a gene-based bioimaging tool to differentiate between LM and LMS in women with suspicious uterine masses. Uterine LMS and LM cannot be differentiated using current imaging techniques. Survivin is increased in LMS, but not in LM. Using Ad-SUR-NIS, we were able to detect NIS in the presence of Survivin. NIS enables the uptake of F18-NaBF_4_ to cells, which leads to the visualization and identification of uterine LMS, but not LM.

This study aimed to evaluate the efficacy and safety of Survivin-Sodium iodide symporter (Ad-Sur-NIS) as a reporter gene biomarker to differentiate between uterine LM from uLMS by positron emission tomography (PET) imaging using LM and LMS xenograft mouse models (Figure 1).

## 2. Materials and Methods

### 2.1. Human Leiomyoma and Human Uterine Leiomyosarcoma Cells

Immortalized human LM cells were cultured in phenol red-free 10% fetal bovine serum Dulbecco’s Modified Eagle Medium: Nutrient Mixture F-12. Human LMS cells (SK-UT1, ATCC^®^ HTB-114^TM^) (ATCC, Manassas, VA, USA) were cultured in ATCC-formulated Eagle’s Minimum Essential Medium with 10% fetal bovine serum.

### 2.2. Reagents

The Ad-Sur-NIS was produced by Vector Biolabs (Malvern, PA, USA). The PET imaging tracer, F18-labeled sodium tetrafluoroborate (F18-NaBF_4_), was purchased from the Cyclotron Facility at the University of Chicago (Chicago, IL, USA). 

### 2.3. Animal Model

The mice were handled according to the IACUC-approved protocol (18-174). Fifty-four nu/nu nude mice were purchased from Charles River. The mice were provided with autoclaved water and a standard natural ingredient diet ad libitum and were maintained in an AAALAC-accredited pathogen-free climate-controlled facility at a 12 h light/dark cycle; 2 × 10^7^ LMS or LM cells were inoculated into the right flank in 1:1 Matrigel and fetal bovine serum (FBS). After tumor development, the animals were randomized and separated into groups: LMS Ad-Sur-NIS, LMS PBS, LM Ad-Sur-NIS, and LM PBS. PBS was used as a vehicle for Ad-Sur-NIS administration.

### 2.4. PET/CT Scan

**Figure 2:**
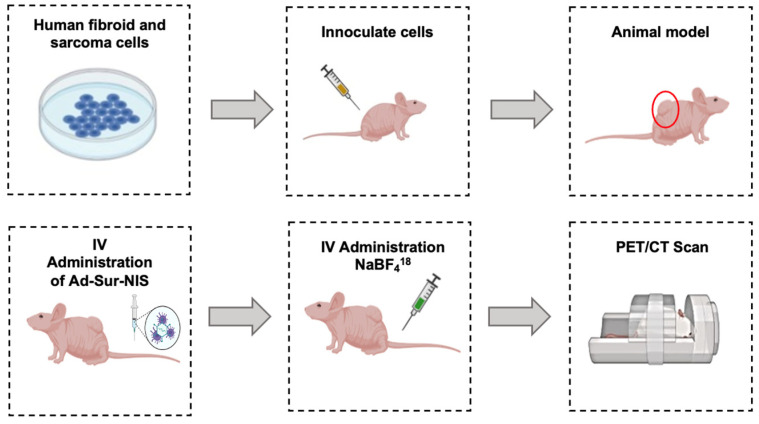
Experimental Design. uLMS and uterine LM cells were cultivated. The cells were inoculated in the right flank of the nude mice. After tumor development, the animals received an intravenous administration of Ad-Sur-NIS or PBS. Then, 24 h later, all the animals received F18-NaBF_4_ through intravenous administration, and PET/CT scans were performed.

A total of 40 animals (LMS Ad-Sur-NIS n = 10, LMS PBS n = 10, LM Ad-Sur-NIS n = 10, and LM PBS n = 10) were imaged with PET/CT using a micro-PET/CT scanner (Trans-PET Discoverist 80, Raycan Technology Co., Ltd., Suzhou, China). Twenty-four hours before the PET/CT imaging, the animals received a dose of either Ad-Sur-NIS (1 × 10^9^ PFU in 0.2 mL/mouse) or PBS (0.2 mL/mouse) through retro-orbital injection. On the day of the PET/CT scan, a dose of 300–400 uCi of F18-NaBF_4_ was given intravenously through a tail-vein injection under isoflurane anesthesia. The anesthesia was continued, and the mice were placed on the sample stage of the PET scanner on a heating pad. Two PET/CT scans were conducted for each mouse, the first immediately, 5 min after injection of F18-NaBF_4_, and the second scan was run after 45 min (Figure 2). Each scan lasted 10 min in static mode, and the mice were taken out of the PET/CT scan machine between the two scans. The PET and CT images were reconstructed using PiSYS software (version 1.3, Raycan Technology Co., Ltd.) associated with the scanner and were exported in DICOM format for analysis.

### 2.5. PET/CT Scan Analysis

Carimas 2.10 (Turku PET Centre, Turku, Finland) was used to analyze the PET images. A 3-dimensional region of interest (ROI) was drawn for the tumor area in each mouse. Corresponding CT images were overlaid with the PET images as an anatomical reference. The ROI was smoothed once before the PET intensities (i.e., the F18 activities) were exported. The standardized uptake value (SUV_max_ or SUV_mean_) for each ROI was calculated by using the formula $SUV=\frac{A/\rho }{D/W}$, where A is the maximum or mean F18-NaBF_4_ activity of the ROI, *ρ* is the density of the mouse (~1 g/mL), D is the total injected dose, and W is the weight of the mouse. SUV_max_ values were used to create the graphics.

### 2.6. Safety Study—H&E Stain and Chemical Metabolic Panel

Fourteen animals were used to determine the safety of Ad-Sur-NIS (LMS Ad-Sur-NIS n = 4, LMS PBS n = 4, LM Ad-Sur-NIS n = 3, LM PBS n = 3). Twenty-four hours after the Ad-Sur-NIS injection, blood (serum) and organ (brain, kidney, liver, lung, heart, ovary, uterus spleen, and tumor) samples were collected. Serum samples were used to evaluate the liver function and a blood chemistry panel was performed by the Biological Laboratories Resource Laboratory Services unit. Organ samples were fixed in 10% buffered formalin for 24 h, then processed and embedded in paraffin, and 5-micron sections were made and stained with H&E. The H&E slides were evaluated by a veterinary pathologist (MCB). 

### 2.7. Statistical Analysis

Data were presented as mean ± standard error (SE). A significant difference was defined as *p* < 0.05. A comparison of the 2 groups was carried out using the parametric Student’s *t*-test for normally distributed data and a nonparametric Mann–Whitney test for not-normally distributed data. A comparison of multiple groups was carried out by analysis of variance (ANOVA) followed by a Tukey post hoc test for normally distributed data parametric distribution and a Kruskal–Wallis test followed by Dunn’s post hoc test for nonparametric not normally distributed data. The statistical analysis was carried out using GraphPad Prism 5 Software.

## 3. Results

### 3.1. Increased Radiotracer Uptake and Decreased Radiotracer Decay Attributable to Ad-Sur-NIS in the LMS Tumors When Compared to LM

The Ad-Sur-NIS was constructed based on our previous results [26], showing that ad-Sur-Luc could differentiate LMS from LM. We selected the NIS to be used as a reporter gene, and its expression can be detected through a PET scan using F18-NaBF_4_ as a radiotracer, allowing Ad-Sur-NIS to be used as a human application in the future.

**Figure 3:**
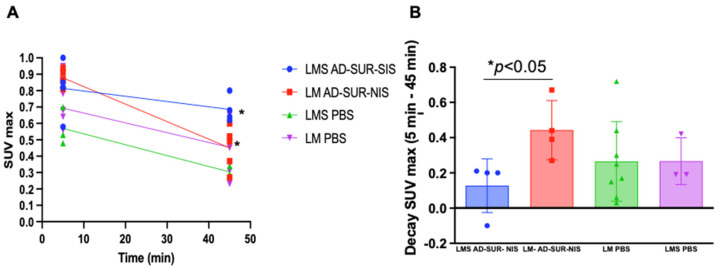
Increased uptake of the F18-labeled tracer attributable to Ad-Sur-NIS in uLMS. (**A**) PET/CT scan identifies uptake time after 5 and 45 min radiotracer administration. At minute 45, a significant difference between LMS Ad-Sur-NIS versus LM Ad-Sur-NIS was *p* < 0.05. (**B**) Decrease in SUV max decay between minutes 5 and 45 in LMS Ad-Sur-NIS compared to LM Ad-Sur-NIS *p* < 0.05.

**Figure 4:**
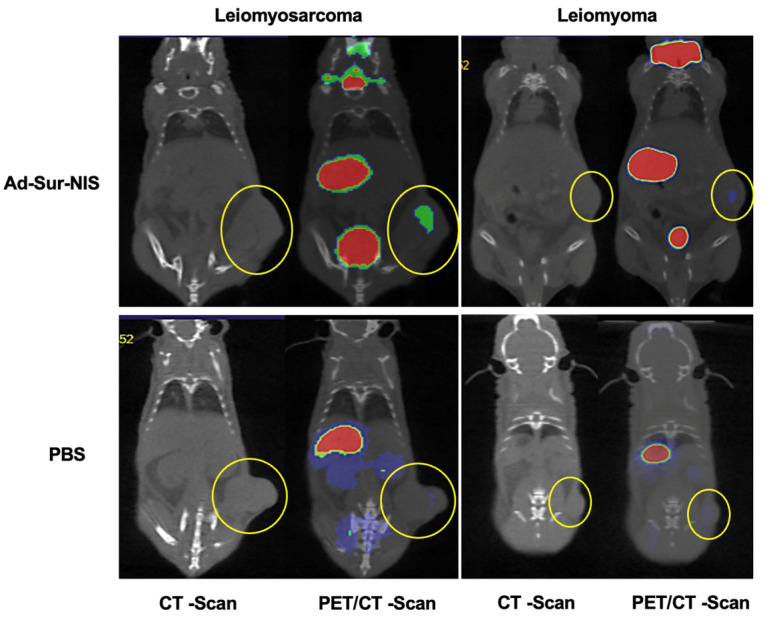
PET/CT images of Ad-Sur-NIS treated mice and control mice (treated with PBS). The PET/CT scans were performed 45 minutes after the F18-NaBF_4_ administration. The tumors are identified in CT images as indicated by the yellow circles. The uptake intensities of F18-NaBF_4_ are “rainbow” color-coded from red (high intensity) to green (medium intensity) to blue (low intensity). While large amounts of F18-NaBF_4_ are presented in the bladder, stomach, and thyroid as intrinsic uptake/distribution, the uptake of F18-NaBF_4_ in LMS is clearly visualized (green color), while the uptake of F18-NaBF_4_ in LM is minimal. Control experiments with mice treated with PBS (instead of Ad-Sur-NIS) indicated no uptake of F18-NaBF_4_ in both LMS and LM. The results indicated that the F18-NaBF_4_ PET/CT scan is capable of differentiating malignant LMS from benign LM.

After the mice PET/CT scanning, we identified no uptake difference among the groups at the early capture time point (5 min after radiotracer administration). However, at the late capture (at minutes 45 after administration), we were able to differentiate uLMS tumors from LM tumors using Ad-Sur-NIS (*p* < 0.05), as demonstrated in Figure 3A,B. Along with these findings, we also found that the use of Ad-Sur-NIS decreased radiotracer decay (comparing early and late capture) in LMS tumors compared to LM tumors (*p* < 0.05), allowing us to further differentiate one condition from the other (Figure 3B and Figure 4).

### 3.2. Evaluation of the Safety of Ad-Sur-NIS

Our study revealed that the administration of Ad-Sur-NIS did not cause differences in a metabolic chemistry panel compared to the PBS group (Table 1). Specifically, we did not find evidence of drug-induced liver injury (Table 2). 

**Figure 5:**
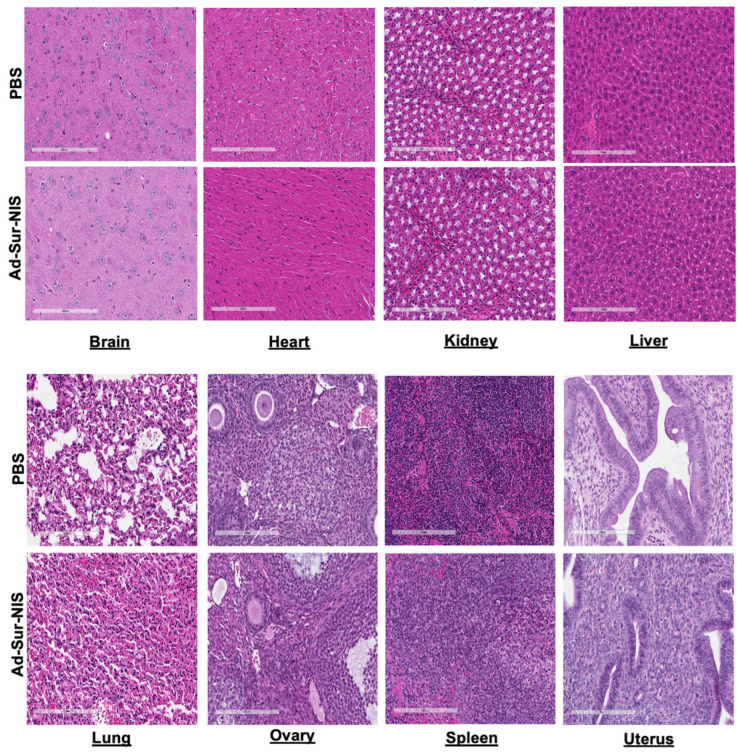
Histologic evaluation of Ad-Sur-NIS. After 24 h of the Ad-Sur-NIS or PBS injection, the various organs, including brain, kidney, liver, lung, heart, ovary, uterus, and spleen, were collected and H&E stain was performed.

We did not observe evidence of tissue injury or morphologic differences between the Ad-Sur-NIS and PBS groups in the brain, heart, kidney, liver, lung, ovary, spleen, and uterus (Figure 5).

## 4. Discussion

In this study, Ad-Sur-NIS in vivo showed that the Survivin promoter could drive NIS expression, specifically in malignant uLMS cells. This is consistent with our previous study showing that human uLMS cells highly expressed the luciferase reporter gene driven by the Survivin promoter compared to benign LM [18]. However, a new probe with a clinical application was needed due to limitations in the use of luciferase as a reporter gene for human application. The human sodium/iodide symporter (NIS), an intrinsic membrane glycoprotein with 13 putative transmembrane domains, plays an essential role in the biosynthesis of thyroid hormones by mediating the active transport of iodide into the thyrocytes [27].

The tumor-specific expression of NIS genes has been identified in many types of tumors, including prostate, colon, and liver cancer, suggesting that NIS expression under the control of tissue-specific promoters could be helpful in diagnostic and therapeutic applications [27,28,29,30]. Notably, adenosine triphosphatase copper ion transporting beta expression is aberrantly upregulated in uLMS cells, and copper sulfate acts as an inhibitor of platinum efflux via the transporter. Combining copper sulfate pretreatment with cisplatin administration exhibited an antitumor effect in mice with uLMS cell xenografts [31]. Using the relevant knowledge, we were able to use Ad-Sur-NIS to distinguish LMS from LM xenografts in nude mice using PET/CT scanning by comparing radiotracer uptake and decay, which may offer a promising non-invasive diagnostic tool to determine malignant LMS from benign LM.

uLMS is a rare and highly aggressive tumor with a 5-year survival of 10–15% for women with metastatic disease. By contrast, LM is benign and common, with up to 70–80% of women developing LM during their lifetime. The differential diagnosis between these two tumors is challenging since the clinical symptoms of both tumors overlap, and more than 50% of women with LMS are initially treated as having LM with potentially inadequate or non-oncology surgery, risking worse outcomes [32]. Although malignant uLMS and benign LM share some common clinical characteristics [33], the molecular signature and biological pathways differ between these two kinds of tumors [34,35,36,37,38]. To overcome the limitations in clinical and radiographic predictors, as well as the lack of reliable serum or urinary biomarkers, the development of a new clinical tool to differentiate LMS from LM in gynecologic practice is urgently needed to select the correct treatment pathway for patients with a uterine mass.

There are several studies evaluating different tools to differentiate LM from LMS. However, the results are debatable, and no evidence is presented that any of these approaches could reliably distinguish LMS from LM [32,39,40,41]. Several imaging modalities, such as MRI, Doppler sonography, and positron emission tomography, have been attempted, but their predictive value still needs to be determined [41,42,43]. Ultrasonography is a standard imaging modality used in the gynecology clinic, but the similar appearances of LMS and LM limit its use for their differential diagnosis [32,44]. Although recent studies have demonstrated that MRI features could help differentiate LMS from LM, its use in LMS diagnosis is challenging because the appearance of LMS in MR images is variable and overlaps with features of degenerated LM [32,45].

The application of PET scans in oncology plays an important role in assessing early diagnosis and pharmacokinetics and pharmacodynamics of novel therapeutics using radiolabeled compounds [46]. As a non-invasive imaging methodology, PET has been utilized with the administration of compounds labeled with radiotracers that are formulated for intravenous injection. Several radionuclides for PET imaging have been used [47].

In this study, we used F18-NaBF_4_ as a nuclide because it has a relatively long half-life, allowing for transport from the production site to the PET centers, high labeling yields, high specific activity, and a high resolution of images [48]. Moreover, the PET radioligand F18-NaBF_4_ was used to image NIS. F18-NaBF_4_ has been shown to increase uptake in known areas with high NIS expression without adverse effects [49]. In contrast to previously used iodides, F18-NaBF_4_ and other F18-labeled iodide analogs have practical radiosynthesis and biochemical properties that allow them to mimic iodide transport by NIS closely [47].

In addition to imaging the LMS and LM, we also examined whether the administration of Ad-Sur-NIS caused any side effects or toxicity. We did not observe any pathological or metabolic changes or indications of liver injury. These studies indicate that Ad-Sur-NIS with the use of F18-NaBF_4_ is safe in our experimental system and suggest that it may be considered a safe, non-invasive diagnostic tool in human application. Moreover, in combination with radioiodine therapy, this strategy has excellent potential for LMS gene therapy.

Notably, the expression of Survivin is detected in human cancers, but not in normal adult tissues. In this study, we used the cancer-specific enhanced Survivin promoter to determine the promoter driving potential of downstream reporter genes. We demonstrate that the F18 NaBF_4_ PET/CT approach can distinguish benign LM from malignant uLMS. According to the “cancer-specific promoters’ hypothesis”, this new approach would provide useful information to triage suspicious lesions in order to distinguish benign tumors from uLMS and other gynecological cancers preoperatively. A potential issue is that using the Ad-Sur-NIS system in uLMS requires phase I assessment. However, similar studies have been reported in nuclear medicine, such as a thyroid scan for the evaluation of suspicious thyroid masses. Therefore, this approach has great potential to be adopted in clinical practice in the future. 

## 5. Conclusions

In conclusion, the F18-NaBF_4_ PET/CT scan using Ad-Sur-NIS is a promising and non-invasion diagnostic tool to distinguish malignant uLMS from benign counterparts. It has great potential to impact the management of suspicious uterine masses, a significant challenge in clinical gynecology. 

## 6. Patents

The results from this manuscript are part of the US2021/0244832A1 patent.

## Figures and Tables

**Table 1:** The chemistry metabolic panel evaluated after the administration of Ad-Sur-NIS or vehicle (PBS).

Parameter	LMS AD-SUR-NIS	LM AD-SUR-NIS	LMS PBS	LM PBS	*p*-Value
Albumin g/dL	3.002 (±0.12)	2.93 (±0.11)	3.41 (±0.15) *	2.88 (±0.075) *	0.025
Alkaline Phosphatase (ALP) U/L	102.25 (±37.31)	102.33 (±21.54)	91.5 (±4.50)	90.33 (±5.85)	0.94
Alanine Aminotransferase (ALT) U/L	34.5 (±8.38)	51.33 (±23.28)	30.5 (±7.35)	35.66 (±12.01)	0.346
Amylase U/L	422.25 (±52.94)	411.66 (±46.09)	416.25 (±63.17)	402.33 (±32.53)	0.0814
Aspartate Aminotransferase (AST) U/L	259.75 (±84.98)	367.33 (±159.2022)	253 (±178.095)	256.66 (±144.68)	0.8157
Urine Nitrogen (BUN) mg/dL	20.25 (±5.29)	25.66 (±2.08)	23.5 (±3.31)	28 (±2.64)	0.3171
Creatinine Kinase U/L	2994.75 (±2379.35)	3251.33 (±1632.798)	1782.75 (±1897.929)	2337.5 (±1546.443)	0.59
Creatinine mg/dL	0.0686 (±0.3538)	0.0153 (±0.124)	0.0724 (±0.0945)	0.0665 (±0.0156)	0.606
Direct Bilirubin mg/dL	0.0275 (±0.015)	0.0166 (±0.0115)	0.03 (±0.008)	0.0266 (±0.0115)	0.47
Gamma Glutamyl Transferase (GGT) U/L	−6.25 (±4.34)	−8.33 (±5.68)	−7 (±3.829)	−6.33 (±5.131)	0.93
Lactate mg/dL	67.95 (±10.51)	57.866 (±8.87) *	93.425 (±12.28) *	41.9 (±3.49)	0.0357
I Phosphorus mg/dL	7.57 (±1.36)	7.13 (±0.64) *	13.45 (±0.50) *	9 (±1.70)	0.0274
Total Bilirubin md/dL	0.3025 (±0.124)	0.29 (±0.167)	0.375 (±0.118)	0.32 (±0.075)	0.7576
Total Protein g/dL	4.795 (±0.21)	4.67 (±0.2497)	5.312 (±0.217) *	4.48 (±0.169) *	0.0206
Globulin g/dL	1.791 (±0.101)	1.738 (±0.137)	1.893 (±0.081) *	1.6088 (±0.074) *	0.047
Alb/ Glob Ratio	2 (±0)	2 (±0)	2 (±0)	2 (±0)	1

* = *p* < 0.05.

**Table 2:** Calculation of the ratio of ALT and ALP to assess the type of drug-induced liver injury.

**Ratio of ALT and ALP**	**LMS–AD-SUR-NIS**	**LM-AD-SUR-NIS**	**LMS PBS**	**LM PBS**
	0.419	0.623	0.414	0.490

ALT = alanine aminotransferase; ALP = alkaline phosphatase; R = ratio; ULN = upper limit of normal. Interpretation: ≥5 in hepatocellular injury, <2 in cholestatic liver injury, between 2 and 5 in mixed hepatocellular/cholestatic liver injury.

## Data Availability

The data are contained in the article.

## Abbreviations

uLMS	Uterine Leiomyosarcoma
LMS	Leiomyosarcoma
LM	Leiomyoma
Ad-Sur-NIS	Adenovirus Survivin-Sodium Iodide Symporter
F18-NaBF_4_	F18-Labeled Sodium Tetrafluoroborate
PET/CT	Positron Emission Tomography/Computed Tomography
FBS	Fetal Bovine Serum
PBS	Phosphate-Buffered Saline
H&E	Hematoxylin and Eosin
SE	Standard Error
SUV	Standardized Uptake Value
ROI	Region of Interest
ALT	Alanine Aminotransferase
ALP	Alkaline Phosphatase
R	Ratio
ULN	Upper Limit of Normal
MRI	Magnetic Resonance Imaging
